# Transition preparation activities among families of youth on the autism spectrum: Preliminary study using repeated assessments across a school year

**DOI:** 10.1371/journal.pone.0231551

**Published:** 2020-04-16

**Authors:** Anne V. Kirby, Marissa L. Diener, Daniel E. Adkins, Cheryl Wright

**Affiliations:** 1 Department of Occupational and Recreational Therapies, College of Health, University of Utah, Salt Lake City, UT, United States of America; 2 Department of Family and Consumer Studies, College of Social and Behavioral Science, University of Utah, Salt Lake City, UT, United States of America; 3 Department of Sociology, College of Social and Behavioral Science, University of Utah, Salt Lake City, UT, United States of America; 4 Department of Psychiatry, School of Medicine, University of Utah, Salt Lake City, UT, United States of America; University of Wollongong, AUSTRALIA

## Abstract

Much is still unknown about the transition to adulthood for youth with autism spectrum disorder (ASD), including what preparation activities best support positive adult outcomes. Parents play a crucial role in the transition planning and preparation process, yet the existing literature lacks detailed information about parent perceptions about transition preparation activities. To examine family transition preparation activities, we conducted a ten-month study of the transition preparation process of 15 families of youth with ASD across an academic year. Youth were ages 14–17 and 93% male. We collected data on transition preparation activity time spent and parent satisfaction over twenty data collection points. We used multi-level modeling to determine longitudinal trajectories of parent-reported preparation for the transition to adulthood based on endorsed transition preparation activities. Findings from this preliminary study revealed that discussions about the future were the most commonly endorsed activities, while social activities were most associated with increased parental perception of transition preparation over time. This study expands understanding of various transition preparation activities engaged in by families of youth with ASD during high school, though research with a larger and more diverse sample is needed to extend findings.

## Introduction

Autism spectrum disorder (ASD) is a neurodevelopmental condition that affects an estimated 1 in 59 children in the United States [1]. The core features of the diagnosis of ASD include social and communication challenges, accompanied by restricted and repetitive behaviors [2]. Despite emphasis on ASD as a childhood disorder, these symptoms persist through adolescence and adulthood, impacting individual and family functioning at all stages of life [3,4]. In particular, the transition to adulthood is known to be a challenging period for individuals with ASD [5]. During adolescence, many families report feeling unprepared for the transition and uncertain of how to support their child to ensure post-secondary success [6,7]. Fittingly, the exit from high school has been likened to “falling off a cliff” for individuals with ASD because of the drastic reduction of supports and services during this time [4,8].

Existing data on post-school outcomes in the US and in other countries (e.g., [9–11]) point to the need for improving transition preparation to facilitate successful transitions to adulthood that align with the priorities of youth and families. Employment rates are low and when individuals with ASD do work it is often for low hours and wages [12,13]. Participation in post-secondary education is also relatively low [14]. In fact, a national study found that over 50% of youth with ASD had no participation in education or employment during their first two years out of high school [15]. Few individuals with ASD live on their own after high school, with large studies suggesting that most adults with ASD continue to reside with their parents [16,17]. Studies suggest that individuals with ASD who have average or above-average cognitive levels (i.e., ASD without intellectual disability [ID]) are particularly at risk during the transition to adulthood, and face significant challenges as adults [16]. Despite their cognitive abilities, they are frequently un- or under-employed and lack skills to live independently, meanwhile receiving fewer supports and services than individuals with ID [16]. One reason for this discrepancy may be that youth with ASD without ID are often working toward a high school diploma and taking general education classes, while those with ID may be spending more time in special education and/or life skills focused classes [5,18].

The important role of parents during the transition process is clear. Yet, evidence suggests that while parents of youth with ASD often have high hopes for the future, they are often uncertain about how to help their youth attain their ideal post-school goals and do not feel supported in this process [19–22]. Despite their uncertainty, however, parents of youth with ASD play an important role in the transition process and often feel transition planning is primarily their responsibility [23]. Parents are highly involved in the day-to-day practices of planning and preparing for adulthood for youth with ASD [7]. Furthermore, certain activities allocated by parents, such as chores, have been shown to positively predict to post-school outcomes like employment [12]. Studies have also shown that parent expectations significantly predict transition preparation activities and post-school independence among youth with ASD [24,25]. However, little is known about which transition preparation activities parents find most helpful in the process. Though one study did find that parents of youth with ASD were unlikely to report that the transition planning provided through the school system was helpful [26].

Most studies on transition either collect cross-sectional data at a single time point—which miss changes over time—or collect longitudinal data with years between data collection—which may make it challenging to understand influences on changes. The period leading up to the transition to adulthood can be quite turbulent with many changes across a single year; however, these changes are not well understood in the existing literature. To begin to address this gap, we conducted a preliminary in-depth study of transition preparation activities as reported by parents of youth with ASD at multiple time points across a single academic year with a small sample. In this brief report, we address the following research questions: (1) What transition-related activities were youth with ASD and their parents most commonly engaged in throughout the course of a high school academic year, and how satisfied were parents with each activity? and (2) How did parent-reported preparedness for transition vary during the school year when accounting for transition activity endorsement over time?

## Methods

For this study, we collected data over the course of an academic year from parents of youth with ASD. The University of Utah Institutional Review Board (IRB) approved all study procedures.

### Participants

Parents of 15 adolescents with ASD participated in the portion of this study described here; adolescents also completed some assessments and a qualitative interview. We recruited a volunteer sample of participants through listservs of Utah-based organizations for parents of youth with disabilities/ASD, at specialty schools, and at local clinics that provide services to this population. To be included, parents needed to report at enrollment that their 14–17 year-old child had received an autism diagnosis from a qualified provider and was anticipating a high school diploma (vs. completion certificate). One parent was a father and the rest were mothers. Youth and family descriptive information is located in Table 1. As is common among youth with ASD, most participants had co-occurring health conditions including anxiety (*N* = 6) and attention-deficit/hyperactivity disorder (*N* = 8), among others. No participants had a diagnosis of intellectual disability, which is likely due to our requirement that they be anticipating receipt of a diploma. Parents consented and youth assented (because of age) to participation in the study—during this process, a member of the research team provided IRB-approved documents and verbal overview of the study to the parent and youth together and invited them to ask questions.

**Table 1:** Participant characteristics.

Youth and Family Characteristics		N (%)
Youth Age: M(SD)		15.5(0.9) years [range: 14–17]
Youth Gender		
	Male	14 (93%)
	Female	1 (7%)
Youth Race: White		15 (100%)
Youth Ethnicity: Hispanic		2 (13%)
Annual Household Income		
	>$29,999	1 (7%)
	$30,000 - $79,999	2 (13%)
	$80,000 - $149,999	8 (53%)
	≥$150,000	4 (27%)
SRS-2 Autism Severity		
	Did not meet ASD cut-off*	4 (27%)
	Mild ASD	4 (27%)
	Moderate ASD	5 (33%)
	Severe ASD	2 (13%)
WASI-II IQ Score: M (SD)		110.8(15.6) [range: 72–139]

SRS-2, Social Responsiveness Scale, Second Edition. ASD, autism spectrum disorder.

*, SRS-2 T-scores of ≤59 are considered within normal limits, however, the manual states “individuals with very mild autistic syndromes may show scores in the upper end of the normal range if they are well adjusted and their adaptive functioning is relatively intact” [26]. All 4 who did not meet for ASD had scores within six T-score points of the cut-off (scores: 53–57).

### Data collection

Data collection occurred at multiple time points. First, parent and youth participants completed an in-person study visit either at the university or their home before the start of the academic year (i.e., July or August). Initial data collection included an assessment battery with parents and youth including the Social Responsiveness Scale, Second Edition (SRS-2; [27]), and the two-subtest version of the Wechsler Abbreviated Scale of Intelligence, Second Edition (WASI-II; [28]), as well as semi-structured interviews with the youth and their parent separately. Interview data is reported in separate manuscripts [29,30]. The SRS-2 and WASI-II were collected for sample description purposes.

Approximately every two weeks throughout the academic year—a total of 16 times—parents received questions via an emailed survey link through REDCap. We selected a biweekly schedule to promote recall and accuracy of responses. The surveys were very brief but elicited information about the types of transition preparation activities parents and youth had engaged in over the past 2 weeks (from a set list with space to add additional activities), the total number of hours spent on those activities, and parents’ reported rating of transition preparation (i.e., *Rate how prepared you currently feel about (child)’s transition out of high school*? reported on a 100-point sliding scale where 1 = *Not at all prepared* and 100 = *Most prepared I could feel*).

Because we expected many of the activities marked biweekly to be repeated over time, and to reduce burden on response time, we asked our additional questions about activities (e.g., satisfaction) less frequently. Specifically, at three times during the academic year (i.e., November, February, and May), the research team followed-up with a data collection telephone call to solicit additional information about the identified transition preparation activities, including details about and their satisfaction with each activity (rated on a scale three point scale: 0 = not satisfied, 1 = somewhat satisfied, 2 = very satisfied).

### Data analysis

We addressed the first research question by examining descriptive statistics. For the second research question, we used a multilevel modeling approach to estimate mixed model growth curve parameters for parent preparedness over the course of the school year. Specifically, we estimated linear mixed models, with random intercept and time slopes specified [31], using Stata 15.1. For this analysis, we removed activities that were very rare (≤ 5 participants; see Table 2). To account for multiple comparisons we used a Bonferroni correction; the multiple testing adjusted significance threshold was *p* = 0.007 [32].

**Table 2:** Preparation activities.

Transition-Related Activities	N (%)	Total Hours	Satisfaction* M (SD)
Discussions about the future	15 (100%)	366.83	1.29 (0.63)
Adolescent received school-based services	12 (80%)	124.50	1.22 (0.71)
Adolescent received community-based services	5 (33%)	33.00	1.67 (0.47)
Received transition-related information from a service provider	7 (47%)	23.25	1.67 (0.47)
Read or listened to transition information	10 (67%)	80.00	1.13 (0.33)
Attended event with transition information	9 (60%)	49.00	1.81 (0.39)
Adolescent engaged in volunteer or paid work	8 (53%)	282.50	1.63 (0.58)
Adolescent participated in social activities	8 (53%)	199.17	1.67 (0.47)

*, Satisfaction was rated on a 3-point scale (i.e., 0 = not satisfied, 1 = somewhat satisfied, 2 = very satisfied).

## Results

Table 2 presents summary data about the number of participants who reported each transition preparation activity type, how many total hours were spent on each activity by the sample, and the average satisfaction rating was for each activity. Having discussions about the future was the most commonly reported transition-related activity, both in terms of the number of participants (N = 15) and the total hours (367). However, satisfaction with those discussions was only moderate. The activities with the highest reported satisfaction were attending events with transition-related information, adolescent participation in social activities, receiving community-based services, and receiving transition-related information from a service provider.

Table 3 contains results of the mixed models. With regards to significant time by activity predictors of preparedness, there were three significant results. At the beginning of the school year, those who reported receiving school-based services had higher perceived preparedness than those who did not, but demonstrated very little change in preparedness; this is in contrast to those who did not receive school-based services, who showed increased preparedness across the year. Similarly, those who did not participate in volunteer or paid work experiences showed more growth in preparedness across the year than those who did. However, participation in social activities was associated with a significant increase in preparedness across the year compared with those who did not report participating in social activities. The types of social activities reported by parents included things like school band and scouting.

**Table 3:** Multilevel (mixed) model growth curve estimates for parent preparedness trajectories.

Variable	Transition-related discussions with youth	Transition-related school-based services	Transition information from service provider	Read or listened to transition information	Attended transition-related events	Youth engaged in paid or unpaid work	Youth engaged in social activities to build skills
Fixed effects							
Time	0.75	1.32***	1.10***	0.88**	1.09***	1.27***	0.66*
Predictor	-4.57	12.05*	2.78	-7.99	5.84	5.93	-16.77***
Time*Predictor	0.37	-1.57**	-0.60	0.62	-0.64	-1.11*	2.38***
Intercept	44.48***	39.98***	41.22***	42.97***	41.05***	40.51***	44.30***
Random effects							
Time	-0.31	-0.16	-0.16	-0.42	-0.25	-0.21	-0.64
Intercept	2.80***	2.73***	2.78***	2.79***	2.77***	2.78***	2.77***
Residual	2.24***	2.20***	2.23***	2.24***	2.24***	2.21***	2.20***
Statistics							
Ll	-722.78	-716.63	-722.17	-721.62	-722.6	-718.08	-712.98
Aic	1459.56	1447.27	1458.34	1457.24	1459.21	1450.15	1439.96
Bic	1482.22	1469.92	1481	1479.9	1481.86	1472.81	1462.61
N	188	188	188	188	188	188	188

*, *p* < 0.007

**, *p* < 0.0014

***, *p* < 0.00014.

## Discussion

This repeated time points study allowed in-depth exploration into transition preparation activities for youth with ASD during an academic year. In this study, parents reported frequent discussions about the future; these were the most commonly endorsed transition activity and ranked second for total time spent. Frequent discussions about the future at home have been shown in prior studies to be a predictor of involvement and participation in transition planning meetings at school which are associated with postsecondary outcomes such as college enrollment [33,34]. Notably, however, the parents in this study reported only moderate satisfaction with these discussions, and these discussions were not predictive of change in parent-reported preparedness in the mixed model. Future work may need to consider ways to best support families to have productive and impactful discussions about the future that facilitate successful transitions to adulthood for youth with ASD.

Social activities were endorsed by about half the participants, but accounted for the highest average time per participant and parents reported relatively high satisfaction. Furthermore, we found using mixed models that social activities were predictive of increased preparation across the year. The endorsed activities were not social skills classes, but real-world social experiences. Though social activities are usually not selected solely for their ability to help youth prepare for adulthood, parents in this study reported that these activities were important preparation for their children. During data collection phone calls, parents explained that these activities helped youth learn about responsibility and working with others, skills that will be useful in future employment. Prior research with stakeholders has identified that real life experiences that foster skill development are positive for transition planning [35].

Our other mixed model findings are less easy to interpret. We found that for both school-based services and work experiences, parents reported greater preparation at the start of the school year but did not show increases associated with those experiences across the year. It may be that those who knew they would be receiving school-based services felt optimistic that the school would be helping to prepare their youth, however, as the year went on, they did not see the progress they anticipated and thus their level preparedness remained static. Prior research has demonstrated parent frustrations with transition planning and school supports in high school, where there are commonly misalignments between what is needed and what is received (e.g., [26,36]).

Regarding volunteer and paid work, we also saw that parents reported higher levels of preparedness at the beginning of the year, but no increases over time. Although most people believe that volunteer and paid work experiences can be valuable for transition preparation, parents may have observed youth having difficulties in those experiences leading them to feel less prepared across the year. It is important to note, however, that prior research has found that employment while in high school is the strongest predictor of post-school employment for youth with severe disabilities [12]. Thus, perhaps parents’ perceptions did not change, but these experiences may still be providing value for transition preparation. Alternately, this result may indicate that youth were not receiving adequate supports during these employment or volunteer experiences. More work is needed in this area to better understand how different transition preparation activities affect long-term outcomes for youth with ASD.

There are a number of limitations to consider for this preliminary study. First, the study is limited by a small sample size that lacked diversity. However, we collected in-depth information across an academic year, providing novel and robust information, and had sufficient power to detect effects using multi-level modeling. Second, there may have been a “testing effect” experienced by participants because of the frequent data collection, such that the experience of being repeatedly contacted by a researcher changed their experiences. Third, the relatively wide age ranges of youth may be viewed as a limitation; however, it may have also served as a strength to include a wide range in an exploratory study. Studies with larger samples are needed to examine age-related differences. Finally, it is important to consider that parent perceptions, as studied here, are distinct from more objective measures of preparedness. However, given the pivotal role parents play during the transition preparation process and the influence of their expectations on activity selection [24] and outcomes [25], their perceptions warrant consideration.

## Conclusions

Our preliminary study of parent-endorsed preparation activities throughout an academic high school year provides an understanding of the various transition preparation activities youth with ASD participate in during their adolescence. Commonly endorsed activities included discussions with youth and receiving school-based services. Whereas, adolescent social activities were more connected with parent satisfaction and linked to an increased sense of preparation. This research can be used to inform future work to understand the transition preparation needs of families of youth with ASD and inform family-focused interventions to support successful transition to adulthood. More research is needed to generate direct applications for clinical practice, however, based on the findings of this study, clinicians may want to especially promote productive family discussions about the future and youth engagement in social activities.

## Supporting information

S1 Appendix (DOCX)
