# Supplementary material for: Transition preparation activities among families of youth on the autism spectrum: Preliminary study using repeated assessments across a school year
Source: PLoS One. 2020 Apr 16;15(4):e0231551. doi: 10.1371/journal.pone.0231551 (PMC7161970; doi:10.1371/journal.pone.0231551)
Supplement: S1 Appendix — (DOCX) [file pone.0231551.s001.docx]

**REDCap Follow-ups**

1. Rate how prepared you currently feel about (youth name)’s transition out of high school:

1 100

Not at all prepared Most prepared I could feel

2. Rate your overall level of stress during the past 2 weeks:

1 100

Not at all stressed Most stressed I could feel

3. Rate your level of worry about (youth name)’s future during the past 2 weeks:

1 100

Not at all worried Most worried I could feel

4. Describe the **one aspect** of (youth name)’s future that you are **most concerned** **about currently**.

5. We would like to know how much time you have spent in the past 2 weeks doing activities to prepare for your child’s transition out of high school. Select the transition-related activities you, (youth name), or others in your family participated in over the past 2 weeks. Fill in how many hours were spent in the past 2 weeks on each activity you selected.

- Discussions about future plans _____hrs
- Received school-based services focused on transition _____hrs
- Received community-based services focused on transition _____hrs
- Received transition-related information from a service provider (such as transition coordinator, vocational rehabilitation) _____hrs
- Read or listened to information related to transition (such as books, websites, podcasts) _____hrs
- Attended event(s) with information related to transition _____hrs
- Adolescent engaged in volunteer work or part-time employment to prepare for transition ____hrs
- Adolescent participated in social activities to prepare for transition _____hrs
- Completed paperwork related to transition (e.g., SSDI paperwork) _____hrs
- Visited potential sites for higher education, employment, housing, etc. _____hrs
- Submitted application for higher education, employment, housing, etc. _____hrs
- Other (please describe) _____________________________________ _____hrs

6. Have any new decisions been made since last study contact about (youth name)’s situation after high school (such as higher education, employment, or housing plans) over the past two weeks? If yes, please describe. No Yes (please describe) ____________________________________

What is the transition-related activity?

Where does it occur?

How was the activity identified?

Why was the activity chosen?

How many hours spent participating (within timeframe)?

Frequency of participation: Ongoing

_______ hours per ____________

Satisfaction with this activity:

0 1 2

Not satisfied Somewhat satisfied Very satisfied

How much more prepared for the future do you feel as a result:

0 1 2

No more prepared Slightly more prepared Significantly more prepared

What is the transition-related activity?

Where does it occur?

How was the activity identified?

Why was the activity chosen?

How many hours spent participating (within timeframe)?

Frequency of participation: Ongoing

_______ hours per ____________

Satisfaction with this activity:

0 1 2

Not satisfied Somewhat satisfied Very satisfied

How much more prepared for the future do you feel as a result:

0 1 2

No more prepared Slightly more prepared Significantly more prepared
